# Effects of grip strengthening combined with scapular stabilization exercise on shoulder muscle strength and functional outcomes in individuals with subacromial pain syndrome: A randomized controlled trial

**DOI:** 10.1097/MD.0000000000050944

**Published:** 2026-10-23

**Authors:** Jae-Hyun Lee, Eunjung Chung, Byoung-Hee Lee

**Affiliations:** a Department of Physical Therapy, Graduate School of Physical Therapy, Sahmyook University, Seoul, Republic of Korea; b Department of Physical Therapy, Andong Science College, andong-si, Republic of Korea; c Department of Physical Therapy, Sahmyook University, Seoul, Republic of Korea.

**Keywords:** grip strength, rotator cuff, shoulder muscle strength, SPADI, subacromial pain syndrome

## Abstract

**Background:**

Subacromial pain syndrome (SAPS) is one of the most common causes of shoulder pain and is frequently associated with rotator cuff dysfunction. Recent evidence suggests a close relationship between hand grip strength and rotator cuff muscle function, indicating that grip-strengthening exercises may enhance shoulder rehabilitation outcomes. To investigate the effects of grip strengthening combined with scapular stabilization exercise on shoulder muscle strength, grip strength, and functional outcomes in individuals with SAPS.

**Methods:**

Thirty-six individuals with SAPS were randomly assigned to an experimental group (scapular stabilization exercise combined with grip strengthening; n = 18) or a control group (scapular stabilization exercise alone; n = 18). Both groups participated in exercise sessions 3 times per week for 4 weeks. Shoulder muscle strength, grip strength, and functional outcomes using the Shoulder Pain and Disability Index were assessed before and after the intervention.

**Results:**

Both groups showed significant improvements in shoulder muscle strength, grip strength, and Shoulder Pain and Disability Index scores (*P* < .001). Significant Time × Group interaction effects were observed for all outcome measures (*P* < .001), indicating greater improvements in the experimental group.

**Conclusions:**

Grip strengthening combined with scapular stabilization exercise may provide additional benefits for improving shoulder muscle strength, grip strength, and functional outcomes in individuals with SAPS.

## 1. Introduction

The shoulder joint has a wide range of motion and plays an essential role in daily activities; however, its mobility also makes it susceptible to pain, injury, and functional impairment.^[1]^ Subacromial pain syndrome (SAPS) is one of the most common causes of shoulder pain encountered in clinical practice and is frequently associated with rotator cuff dysfunction.^[2]^ SAPS encompasses several shoulder conditions, including rotator cuff tendinopathy, subacromial bursitis, and subacromial impingement syndrome.^[1,3]^ Its etiology is multifactorial and may involve rotator cuff dysfunction, muscular imbalance, and altered scapular kinematics.^[1,4]^ Individuals with SAPS commonly experience shoulder pain, muscle weakness, and functional limitations, which may negatively affect activities of daily living and quality of life.^[1,3]^

Exercise therapy is a cornerstone of conservative management for SAPS and aims to improve shoulder muscle performance and functional ability.^[2,5]^ In particular, scapular stabilization exercises have been widely used to improve scapular control and shoulder function by promoting the activation of the serratus anterior, lower trapezius, and rotator cuff muscles.^[3,6]^ Previous systematic reviews have reported beneficial effects of scapular-focused rehabilitation on pain and shoulder function in individuals with SAPS.^[6,7]^ However, despite the established role of scapular stabilization exercises, the optimal combination of exercise strategies for further improving shoulder muscle performance and functional outcomes remains unclear.^[7]^ Therefore, identifying additional exercise components that can be easily incorporated into conventional scapular stabilization programs may be clinically meaningful.

Grip strength has recently received attention as a potential factor associated with shoulder muscle function. Previous research has demonstrated a significant relationship between hand grip strength and rotator cuff function.^[8]^ Moreover, forceful handgrip tasks have been shown to increase activation of the shoulder musculature, suggesting that handgrip activity may influence proximal shoulder muscle recruitment through the upper-extremity kinetic chain.^[9,10]^ Grip strength has also been proposed as an indicator of shoulder function and rehabilitation outcomes.^[11]^ These findings suggest that grip-strengthening exercises may provide a simple and accessible approach for increasing neuromuscular demand within the upper extremity during shoulder rehabilitation.

Although previous studies have demonstrated associations between grip strength and shoulder muscle function and have reported increased shoulder muscle activation during forceful handgrip tasks,^[8–10]^ evidence regarding the effects of incorporating grip-strengthening exercises into a scapular stabilization program in individuals with SAPS remains limited. In particular, it remains unclear whether adding grip-strengthening exercises to conventional scapular stabilization exercise provides additional benefits for shoulder muscle strength, grip strength, and functional outcomes.

Therefore, this study aimed to investigate the effects of grip-strengthening exercises combined with scapular stabilization exercises on shoulder muscle strength, grip strength, and functional outcomes in individuals with SAPS. We hypothesized that the combined intervention would result in greater improvements in shoulder muscle strength, grip strength, and functional outcomes than scapular stabilization exercise alone.

## 2. Methods

### 2.1. Setting and participants

This randomized controlled trial was conducted at S Hospital in Chuncheon, Gangwon State, Republic of Korea. Participants were recruited from patients diagnosed with SAPS who visited the outpatient rehabilitation clinic. The intervention was conducted over a 4-week period beginning in May 2025.

The required sample size was calculated using G*Power software (version 3.1.9.7; Heinrich Heine University, Düsseldorf, Germany). Based on a repeated-measures analysis of variance (ANOVA), with an effect size (f) of 0.50, a significance level of 0.05, and a statistical power of 0.80, a minimum sample size of 36 participants was required. To account for potential dropouts, 96 individuals were initially screened. Of these, 56 were excluded because they were outside the eligible age range (n = 11), had symptom duration of <3 months (n = 14), or declined to participate (n = 31). Consequently, 40 participants underwent baseline assessment and met the eligibility criteria.

Eligible participants were adults aged 18 to 50 years who had experienced SAPS-related symptoms for at least 3 months, demonstrated positive findings on at least 3 of 5 clinical tests for SAPS, had no other musculoskeletal disorders affecting the upper extremity, and were able to understand and follow study instructions. The 5 clinical tests included the Hawkins–Kennedy test, Empty Can test, Painful Arc test, Neer test, and External Rotation Resistance test. The use of a cluster of these tests has been recommended to improve diagnostic accuracy for rotator cuff–related shoulder disorders and SAPS.

Participants were excluded if they had a history of other shoulder pathologies, bilateral shoulder pain, previous shoulder surgery, a symptom duration of <3 months, or had received a corticosteroid injection to the affected shoulder within the previous year.

All participants received a detailed explanation of the study procedures and provided written informed consent before participation. This study was approved by the Institutional Review Board of Sahmyook University (SYU 2024-05-038-010) and was registered with the Clinical Research Information Service (KCT0010308). All procedures were conducted in accordance with the ethical principles of the Declaration of Helsinki.

### 2.2. Experimental procedure

Following the baseline assessment, eligible participants were randomly allocated to either the experimental or control group using the Research Randomizer program (http://www.randomizer.org/). The group allocation was then communicated to the designated therapist, who was responsible for implementing the assigned intervention. This procedure was used to minimize potential selection bias. A total of 40 eligible participants were initially assigned to the experimental group (n = 20) or control group (n = 20). During the intervention, 2 participants from each group withdrew, resulting in 18 participants in each group for the final analysis. No adverse events related to the interventions were reported during the study period.

Outcome measures were assessed before and after the 4-week intervention and included shoulder external rotator strength, shoulder internal rotator strength, grip strength, and shoulder function assessed using the Shoulder Pain and Disability Index (SPADI). The overall study procedure is presented in Figure 1.

**Figure 1.**
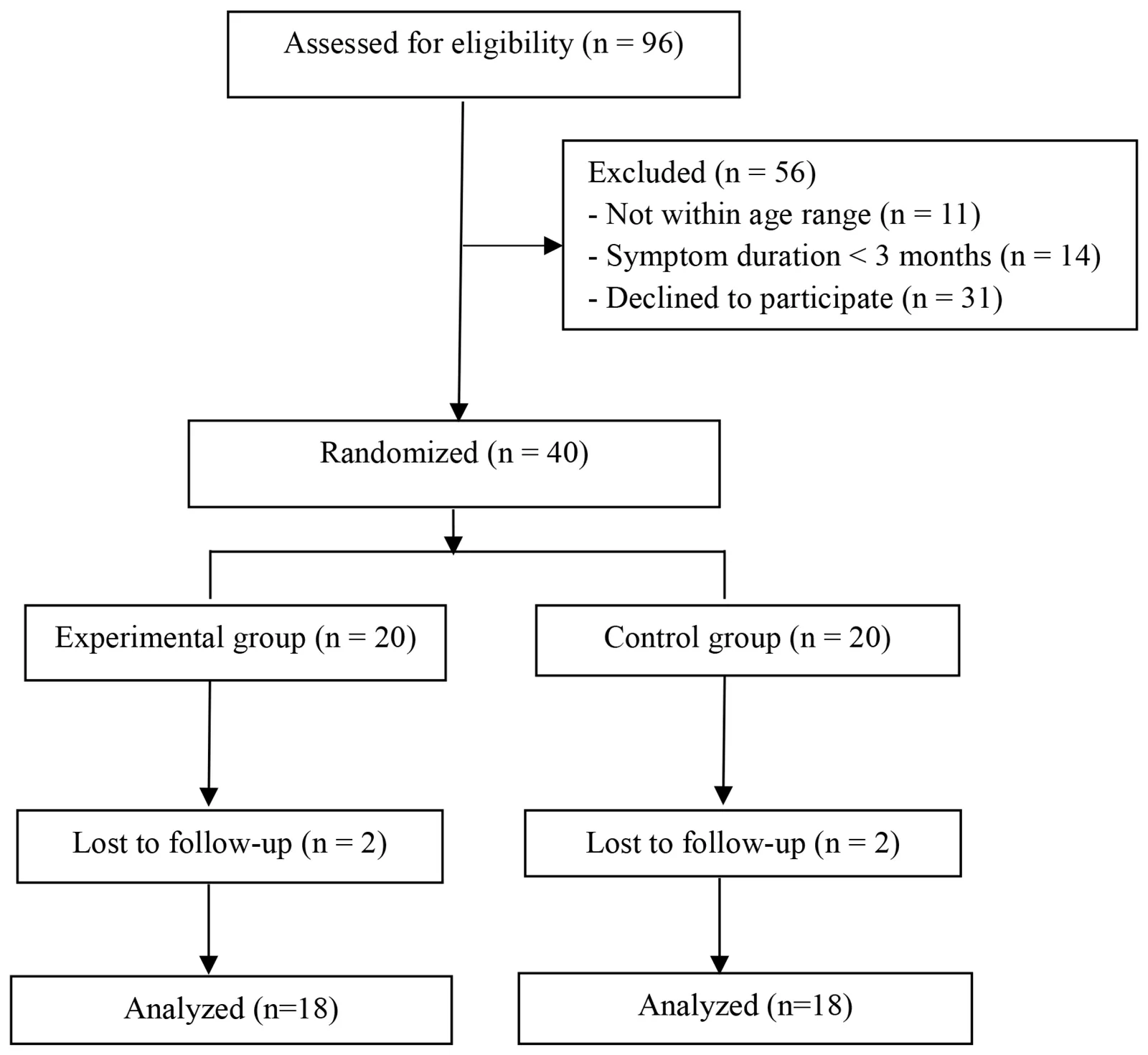
Flow diagram of participants through the study.

### 2.3. Intervention

Both groups participated in supervised exercise sessions 3 times per week for 4 weeks. Before each session, participants performed a standardized 5-minute stretching program consisting of sleeper stretching, posterior shoulder stretching, and pectoralis stretching.

The experimental group performed grip-strengthening exercises in addition to scapular stabilization exercises, whereas the control group performed only scapular stabilization exercises. The total intervention duration was 40 minutes for the experimental group (20 minutes of grip-strengthening exercise and 20 minutes of scapular stabilization exercise) and 20 minutes for the control group (20 minutes of scapular stabilization exercise). All exercise sessions were supervised by a physical therapist to ensure adherence to the prescribed exercise protocol and proper exercise performance.

#### 2.3.1. Grip strengthening exercise

Grip strengthening exercises were performed on the affected side using a hand gripper and a rubber ball. The exercise program was progressively modified over the 4-week intervention period by changing the shoulder position and increasing the exercise volume. In the first week, the exercise was performed with the shoulder positioned at 30° of abduction. The shoulder position was progressed to 60° of abduction in the second week and 90° of abduction in the third week. In the fourth week, the exercise was performed with the shoulder at 90° of abduction combined with 90° of external rotation.

Participants performed 10 repetitions per set, and the number of sets was progressively increased according to the exercise protocol. A 1-minute rest interval was provided between sets. The resistance of the hand gripper was adjusted according to the participant’s condition. The exercise was performed under the supervision of a physical therapist, who provided instructions regarding the prescribed shoulder position and exercise technique. Detailed exercise progression is presented in Table 1, and representative exercise positions are shown in Figure 2.

**Table 1:** Grip strengthening exercise program.

Wk	Shoulder position	Exercise	Volume
1	30° shoulder abduction	Hand gripper + rubber ball	10 repetitions × 3 sets
2	60° shoulder abduction	Hand gripper + rubber ball	10 repetitions × 4 sets
3	90° shoulder abduction	Hand gripper + rubber ball	10 repetitions × 5 sets
4	90° shoulder abduction + 90° external rotation	Hand gripper + rubber ball	10 repetitions × 6 sets

Participants performed grip strengthening exercises using a hand gripper and a rubber ball. A 1-minute rest interval was provided between sets. The total exercise duration was 20 minutes per session.

**Figure 2.**
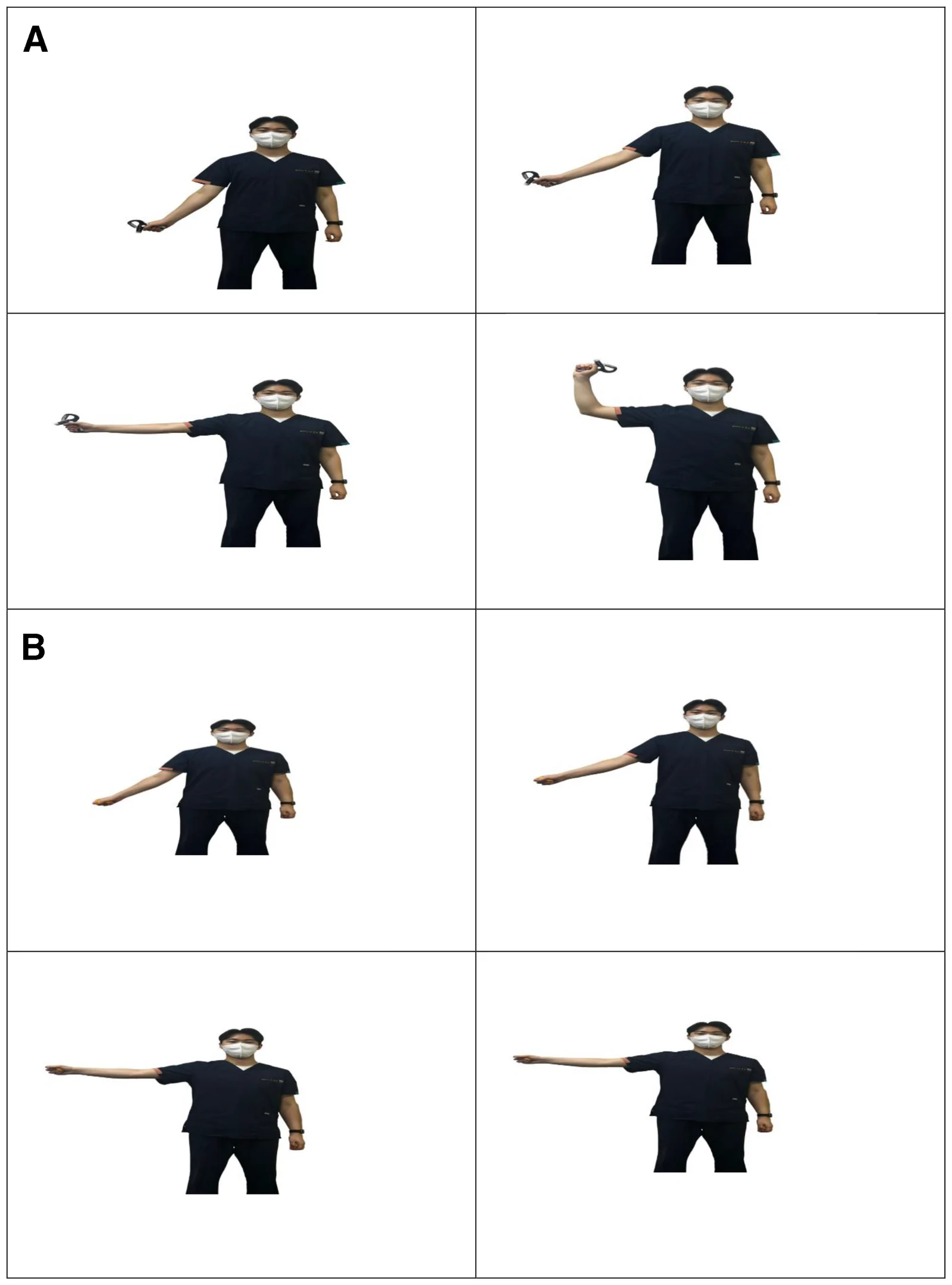
Representative grip-strengthening exercises.

#### 2.3.2. Scapular stabilization exercise

Scapular stabilization exercises were performed by both groups and consisted of rotator cuff strengthening, serratus anterior strengthening, and proprioceptive neuromuscular facilitation (PNF)-based exercises. The exercise program included side-lying external/internal rotation, tubing external/internal rotation, wall slide, pushup plus, and PNF D2 pattern exercises.

All exercises were performed for 10 repetitions and 3 sets, with a 30-second rest interval between sets. Exercise intensity and difficulty were adjusted according to the participant’s condition while maintaining the prescribed exercise technique. The scapular stabilization exercise program was performed for approximately 20 minutes per session. Detailed exercise descriptions are presented in Table 2, and representative exercises are illustrated in Figure 3.

**Table 2:** Scapular stabilization exercise program.

Exercise	Description
Side-lying external/internal rotation	Internal and external rotation exercises performed in a side-lying position with the elbow flexed to 90°.
Tubing external/internal rotation	Internal and external rotation exercises using an elastic resistance band with the elbow maintained at 90° flexion.
Wall slide	Bilateral wall-slide exercise performed while maintaining scapular upward rotation.
pushup plus	Scapular protraction exercise performed in a quadruped position.
PNF D2 pattern	Diagonal upper-extremity movement pattern emphasizing shoulder flexion, abduction, and external rotation.

All exercises were performed for 10 repetitions × 3 sets.

A 30-second rest interval was provided between sets.

The total exercise duration was 20 minutes per session.

PNF = proprioceptive neuromuscular facilitation.

**Figure 3.**
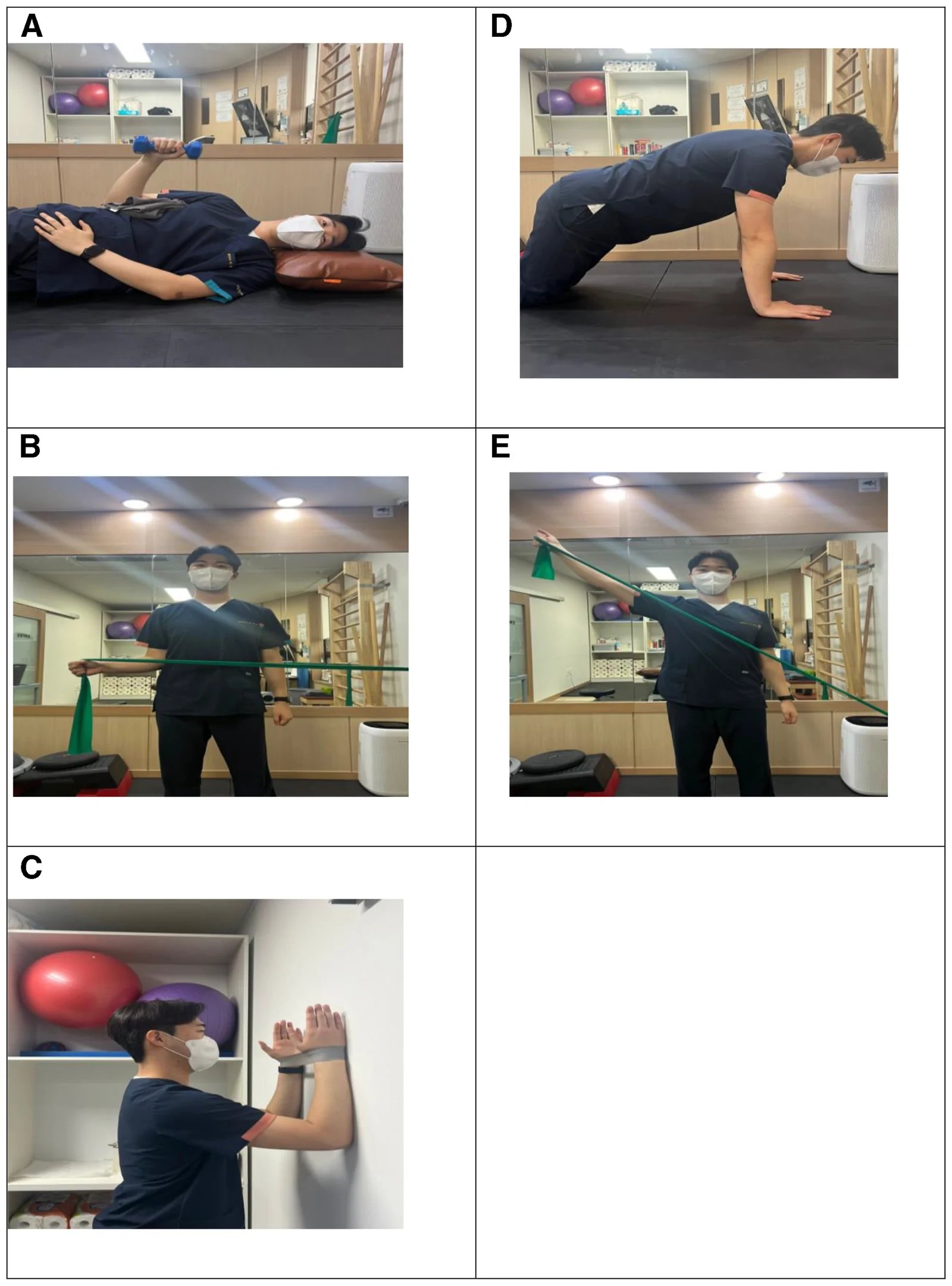
Representative scapular stabilization exercises.

### 2.4. Outcome measures

Outcome measures were assessed before and after the 4-week intervention period in an independent treatment room at S Hospital. The 4 outcome measures were shoulder external rotator strength, shoulder internal rotator strength, grip strength, and shoulder function assessed using the SPADI.

To minimize measurement bias, outcome assessments and interventions were performed by different physical therapists using a single-blind design. All measurements were conducted by licensed physical therapists with more than 3 years of clinical experience who were trained in the use of the assessment instruments and standardized measurement procedures. The same assessment procedures and measurement equipment were used for the pre- and post-intervention assessments. Each measurement was repeated with sufficient rest intervals to minimize the influence of fatigue, and the mean value of the repeated measurements was used for statistical analysis.

#### 2.4.1. Shoulder muscle strength

Shoulder muscle strength was assessed using a digital handheld dynamometer (Commander PowerTrack II Dynamometer; J-Tech Medical, Midvale). Isometric strength of the shoulder external rotators and internal rotators was measured with the participant seated, the elbow flexed to 90°, the forearm in a neutral position, and the arm positioned at the side of the trunk.

Participants were instructed to exert maximal force against the examiner’s resistance for 5 seconds. For each measurement, the examiner provided standardized instructions and resistance while maintaining the participant’s prescribed testing position. Each measurement was performed 3 times with a 30-second rest interval between trials, and the mean value was recorded. Muscle strength was expressed in Newtons (N). The handheld dynamometer has demonstrated good reliability for measuring shoulder muscle strength.

#### 2.4.2. Grip strength

Grip strength was measured using a digital hand dynamometer (T.K.K. 5401; Takei Scientific Instruments Co., Niigata, Japan). Participants stood with their feet shoulder-width apart, their elbow fully extended, and their forearm in a neutral position. They were instructed to exert maximal grip force for 2 seconds.

Following 1 practice trial, 2 measurements were obtained with a 30-second rest interval between trials. Standardized verbal instructions were provided during the measurement, and the mean value of the 2 measurements was used for analysis. Grip strength was expressed in kilograms (kg). The hand dynamometer has demonstrated excellent reliability for grip strength assessment.^[12]^

#### 2.4.3. Shoulder function

Shoulder function was assessed using the SPADI, a self-reported questionnaire designed to evaluate shoulder pain and disability.^[13]^ The SPADI consists of 13 items divided into 2 subscales: a pain subscale (5 items) and a disability subscale (8 items).

Each item is scored using a visual analog scale, and the total score is converted to a percentage ranging from 0 to 100, with higher scores indicating greater pain and disability. The SPADI has demonstrated excellent reliability and validity in individuals with shoulder disorders.^[14]^ The same standardized instructions were provided to all participants when completing the questionnaire.

### 2.5. Statistical analysis

All statistical analyses were performed using SPSS Statistics for Windows, version 30.0 (IBM Corp., Armonk). Descriptive statistics are presented as mean ± standard deviation (SD). The normality of data distribution was assessed using the Shapiro–Wilk test.

Independent t-tests were used to compare baseline characteristics between groups. Paired t-tests were performed to evaluate within-group changes from pre- to post-intervention. A 2-way repeated-measures analysis of variance (ANOVA) was conducted to examine the effects of time (pre-intervention vs post-intervention), group (experimental vs control), and the time × group interaction. The time × group interaction was used to determine whether the changes in outcome measures over time differed between the experimental and control groups.

Statistical significance was set at *P* < .05. Effect sizes were calculated using Cohen’s *d* with corresponding 95% confidence intervals (CIs) to evaluate the magnitude of the intervention effects. Effect sizes were interpreted according to the criteria proposed by Cohen.^[15]^

## 3. Results

### 3.1. Baseline characteristics

A total of 36 participants completed the study and were included in the final analysis, with 18 participants in the experimental group and 18 participants in the control group. No significant differences were observed between the groups in baseline demographic and anthropometric characteristics, including sex distribution, age, height, weight, and body mass index (all *P* > .05). Detailed baseline characteristics are presented in Table 3.

**Table 3:** Baseline characteristics of the participants.

Characteristic	Experimental group (n = 18)	Control group (n = 18)	*P*-value
Sex (Male/Female), n	9/9	11/7	.737
Age (yr)	41.44 ± 7.50	36.44 ± 7.39	.052
Height (cm)	168.94 ± 9.71	170.33 ± 9.02	.660
Weight (kg)	64.78 ± 10.25	68.44 ± 13.16	.358
BMI (kg/m^2^)	22.61 ± 2.20	23.38 ± 2.82	.368

Values are presented as mean ± standard deviation.

BMI = body mass index.

### 3.2. Effects of the intervention on outcome measures

Both groups demonstrated significant improvements in shoulder muscle strength, grip strength, and shoulder function following the intervention (all *P* < .001). Significant time × group interaction effects were observed for external rotator strength, internal rotator strength, grip strength, and SPADI score, indicating that the magnitude of change over time differed significantly between the experimental and control groups.

For shoulder muscle strength, both external and internal rotator strength increased significantly over time, with significant time × group interaction effects. No significant main effect of group was observed for either external or internal rotator strength.

Grip strength significantly increased in both groups, with a significant main effect of group and a significant time × group interaction, indicating a greater improvement in the experimental group.

SPADI scores decreased significantly in both groups, indicating improved shoulder pain and disability. A significant time × group interaction was observed, whereas the main effect of group was not significant.

The between-group effect sizes based on pre–post change scores were large for external rotator strength (Cohen’s *d* = 2.02, 95% CI 1.20–2.82), internal rotator strength (d = 2.09, 95% CI 1.26–2.90), grip strength (*d* = 9.07, 95% CI 6.82–11.30), and SPADI score (*d* = 1.37, 95% CI 0.63–2.09). Detailed numerical results and statistical analyses are presented in Table 4.

**Table 4:** Changes in muscle strength and functional outcome following the intervention (N=36).

Outcome	Experimental (n = 18)	Control (n = 18)	Time F(*P*)	Group F(*P*)	Time × Group F(*P*)	Cohen’s *d* (95% CI)
External rotator strength (N)	6.52 ± 0.90 → 10.18 ± 0.93	7.24 ± 1.14 → 9.91 ± 1.18	1475.456 (<.001)	0.426 (.519)	36.617 (<.001)	2.02(1.20→ 2.82)
Internal rotator strength (N)	7.71 ± 0.91 → 11.92 ± 1.18	8.54 ± 1.19 → 11.29 ± 1.39	891.951 (<.001)	0.067 (.797)	39.211 (<.001)	2.09(1.26→2.90)
Grip strength (kg)	26.01 ± 2.79 → 41.04 ± 3.10	24.74 ± 2.49 → 29.82 ± 2.22	3021.689 (<.001)	51.104 (<.001)	739.773 (<.001)	9.07(6.82→11.30)
SPADI score	42.00 ± 7.32 → 18.83 ± 6.47	37.11 ± 7.77 → 17.22 ± 7.51	2919.824 (<.001)	1.838 (.184)	16.922 (<.001)	1.37(0.63→2.09)

Values are presented as mean ± standard deviation. Cohen’s d represents the between-group effect size based on pre–post change scores, with corresponding 95% confidence intervals (CIs).

SPADI = shoulder pain and disability index.

## 4. Discussion

### 4.1. Effects on shoulder muscle strength

The purpose of this study was to investigate whether adding grip-strengthening exercises to scapular stabilization exercises could provide additional benefits for shoulder muscle strength, grip strength, and shoulder function in individuals with SAPS. Both groups demonstrated significant improvements in the outcome measures following the 4-week intervention. In addition, significant time × group interaction effects were observed for external rotator strength, internal rotator strength, grip strength, and SPADI score, indicating that the magnitude of change over time differed between the 2 intervention groups.

The significant improvements in external and internal rotator strength observed in both groups are consistent with previous evidence supporting scapular stabilization exercise as an effective intervention for individuals with SAPS.^[3,6,7]^ Scapular-focused exercise may improve scapular muscle control and shoulder muscle performance, thereby contributing to improved shoulder function.^[3,6,7]^ In the present study, both groups performed the same scapular stabilization exercise program, which included rotator cuff strengthening, serratus anterior strengthening, and PNF-based exercises. These exercises may have contributed to the observed improvements in shoulder rotator strength.

More importantly, the significant time × group interactions for both external and internal rotator strength suggest that the changes in shoulder muscle strength differed between the 2 groups. One possible explanation is that the addition of grip-strengthening exercise may have increased proximal shoulder muscle recruitment during forceful handgrip activity. Previous studies have demonstrated that forceful handgrip tasks increase shoulder muscle activation,^[9,10]^ and changes in hand grip strength have been reported to correlate with shoulder rotator cuff function.^[8]^ These findings support the concept that handgrip activity is not restricted to the distal muscles of the hand and forearm but may involve coordinated activation of the proximal shoulder musculature.

The progressive shoulder positioning used during grip-strengthening exercise in the present study may also have increased the stabilization demands placed on the shoulder complex. As grip force was generated while the shoulder was positioned at progressively greater degrees of abduction and external rotation, participants may have required greater proximal muscle control to maintain the prescribed shoulder position. This may partly explain the greater changes in shoulder rotator strength observed in the experimental group. However, because muscle activation or scapular kinematics was not directly measured in the present study, this proposed neuromuscular mechanism should be interpreted as a possible explanation rather than a confirmed causal mechanism.

### 4.2. Effects on grip strength

Grip strength significantly improved in both groups, with greater improvement observed in the experimental group. The greater improvement in the experimental group is expected because grip-strengthening exercise was directly incorporated into its intervention. More importantly, the finding demonstrates that a structured grip-strengthening program can improve grip strength when incorporated into a shoulder rehabilitation program.

The relationship between grip strength and shoulder function may provide an additional rationale for including grip-strengthening exercise in shoulder rehabilitation. Horsley et al ^[8]^ reported a significant relationship between changes in hand grip strength and shoulder rotator cuff function, suggesting that grip strength is functionally associated with shoulder muscle performance. In addition, Liu et al ^[11]^ reported that preoperative handgrip strength was associated with early postoperative shoulder function in patients undergoing arthroscopic rotator cuff repair. Although the population and clinical context of Liu et al ^[11]^ differed from those of the present study, these findings support the potential relevance of grip strength to overall shoulder function.

The present study therefore extends previous observational evidence by examining grip strengthening as an active component of a shoulder rehabilitation program in individuals with SAPS. Nevertheless, the present study cannot determine whether the improvement in grip strength directly contributed to the improvement in shoulder muscle strength or shoulder function. Further studies using mediation analyses or direct assessment of neuromuscular activation may help clarify the relationship between grip strengthening and proximal shoulder function.

### 4.3. Effects on shoulder function

SPADI scores improved significantly in both groups following the intervention, indicating reductions in shoulder pain and disability. This finding is consistent with previous evidence supporting exercise-based rehabilitation for SAPS ^[4,5]^ and with studies reporting beneficial effects of scapular-focused rehabilitation on pain and shoulder function.^[6,7]^

The significant time × group interaction observed for SPADI suggests that the pattern of change in shoulder pain and disability differed between the 2 groups. However, because the main effect of group was not significant, the findings should not be interpreted as demonstrating clear overall superiority of the combined intervention. Rather, the results suggest that adding grip-strengthening exercise may influence the magnitude of functional improvement when combined with scapular stabilization exercise.

The improvement in shoulder function may be related to the improvement in shoulder muscle performance observed after the intervention. Increased rotator cuff strength and improved scapular muscle control may facilitate more efficient shoulder movement and reduce functional limitations during daily activities.^[3,6,7]^ In addition, the integration of grip activity with shoulder stabilization may have increased the functional demands placed on the upper extremity. However, because the present study did not directly measure scapular kinematics, muscle activation, or movement coordination, the specific mechanism underlying the improvement in SPADI scores cannot be determined from the present findings.

### 4.4. Clinical implications and interpretation

The findings of this study have several potential clinical implications for the rehabilitation of individuals with SAPS. Both intervention programs were associated with improvements in shoulder external and internal rotator strength and shoulder function, while the experimental group also demonstrated greater improvement in grip strength. These findings suggest that grip-strengthening exercise may be incorporated as an adjunct to scapular stabilization exercise when rehabilitation goals include both shoulder muscle performance and grip strength.

From a practical perspective, the grip-strengthening program used in this study required relatively simple equipment, including a hand gripper and a rubber ball, and was performed on the affected side with progressive changes in shoulder position. Therefore, this type of exercise may be relatively easy for clinicians to incorporate into an existing rehabilitation program without requiring specialized equipment. The progressive positioning of the shoulder also provides an opportunity to integrate distal grip-force production with proximal shoulder stabilization during exercise.

Clinically, the observed improvements in shoulder rotator strength may have potential relevance to activities requiring shoulder stabilization and force production. Similarly, the observed improvement in grip strength may have potential relevance to tasks requiring sustained or forceful hand use. The reduction in SPADI scores indicates improvement in patient-reported shoulder pain and disability. However, these findings should be interpreted as changes observed following the intervention rather than evidence of established clinical significance.

However, the clinical findings should be interpreted cautiously. Statistical significance does not necessarily indicate that the magnitude of change exceeded an established minimal clinically important difference (MCID), and the present study was not specifically designed to determine MCID-based clinical responsiveness. In addition, the experimental group performed an additional 20 minutes of exercise per session compared with the control group. Therefore, the observed between-group differences may reflect both the specific contribution of grip-strengthening exercise and the greater total exercise volume. Accordingly, the present findings support the potential clinical usefulness of grip-strengthening exercise as an adjunct to scapular stabilization exercise, rather than demonstrating that grip strengthening alone produces superior clinical outcomes.

### 4.5. Limitations

Several limitations should be considered when interpreting the findings of this study. First, the sample size was relatively small, which may limit the generalizability of the findings to the broader population of individuals with SAPS. To minimize the potential influence of insufficient sample size, an a priori sample size calculation was performed using G*Power, and the calculated sample size was considered during participant recruitment. Nevertheless, future studies with larger samples are needed to confirm the present findings and improve their generalizability.

Second, the intervention period was limited to 4 weeks, and no follow-up assessment was performed. Therefore, the long-term maintenance of the observed improvements could not be determined. To improve consistency in treatment exposure, both groups participated in supervised exercise sessions 3 times per week for 4 weeks under a standardized intervention schedule. However, longer-term randomized controlled trials with follow-up assessments are needed to determine whether the effects are maintained after the intervention has ended.

Third, the experimental group performed an additional 20 minutes of grip-strengthening exercise per session, whereas the control group performed only 20 minutes of scapular stabilization exercise. Therefore, the specific effect of grip-strengthening exercise cannot be completely separated from the effect of the greater total exercise volume. This limitation was considered when interpreting the between-group differences, and the findings were interpreted as supporting the potential benefit of adding grip-strengthening exercise to scapular stabilization exercise rather than demonstrating an isolated effect of grip strengthening. Future studies should use an equal-duration control intervention to determine the independent contribution of grip-strengthening exercise.

Finally, although standardized measurement procedures were used, the study did not directly assess scapular kinematics, muscle activation, or neuromuscular coordination. Therefore, the physiological mechanisms underlying the observed improvements cannot be confirmed. To minimize measurement-related bias, outcome assessments were performed by trained physical therapists using standardized procedures, and the outcome assessor was separate from the therapist who provided the intervention. Future studies incorporating electromyography and motion analysis may help clarify the mechanisms underlying the effects of grip-strengthening exercise.

## 5. Conclusion

Grip-strengthening exercises combined with scapular stabilization exercises resulted in significant improvements in shoulder muscle strength, grip strength, and shoulder function in individuals with SAPS. These findings suggest that grip-strengthening exercises may be considered a useful adjunct to conventional scapular stabilization programs and may contribute to improved upper-extremity function in patients with SAPS.

## Author contributions

**Conceptualization:** Jae-Hyun Lee, Byoung-Hee Lee.

**Investigation:** Jae-Hyun Lee.

**Methodology:** Jae-Hyun Lee, Eunjung Chung.

**Resources:** Jae-Hyun Lee, Byoung-Hee Lee.

**Data curation:** Eunjung Chung, Byoung-Hee Lee.

**Formal analysis:** Eunjung Chung.

**Software:** Eunjung Chung.

**Project administration:** Byoung-Hee Lee.

**Supervision:** Byoung-Hee Lee.

**Validation:** Byoung-Hee Lee.

**Visualization:** Byoung-Hee Lee.

**Writing – original draft:** Jae-Hyun Lee, Byoung-Hee Lee.

**Writing – review & editing:** Eunjung Chung.
